# Novel Hg^**2+**^-Selective Signaling Probe Based on Resorufin Thionocarbonate and its *μ*PAD Application

**DOI:** 10.1038/s41598-019-40169-6

**Published:** 2019-03-04

**Authors:** Myung Gil Choi, So Young Park, Ka Young Park, Suk-Kyu Chang

**Affiliations:** Department of Chemistry, Chung-Ang University, Seoul, 06974 Republic of Korea

## Abstract

In this study, a novel resorufin thionocarbonate-based Hg^2+^-selective signaling probe (**RT**) for microfluidic paper-based analytical device (*μ*PAD) applications is reported. The designed probe, **RT**, was readily synthesized by the one-step reaction of resorufin with phenyl thionochloroformate. The **RT** probe displayed a prominent color change from yellow to pink and a marked turn-on fluorescence signaling behavior exclusively toward the Hg^2+^ ion. The signaling of **RT** was due to Hg^2+^-induced hydrolysis of the phenyl thionocarbonate moiety to form the parent resorufin dye, which restored its spectroscopic properties. In addition, **RT** exhibited the Hg^2+^-selective signaling behavior without interference by coexisting environmentally relevant metal ions. The detection limit for Hg^2+^ in simulated wastewater samples was estimated to be 5.8 × 10^−8^ M. In particular, an **RT**-equipped *μ*PAD prepared using a wax printing technique enabled simple and convenient determination of Hg^2+^ ions in simulated wastewater samples, with a detection limit of 5.9 × 10^−6^ M.

## Introduction

Mercury, which is released into the environment as vapor or inorganic and organic mercurial species, has been reported to cause serious damage to human health^1^. It has harmful impacts on the respiratory system, causing chest pain, cough, dyspnea, and hemoptysis^2^, and damages the nervous system, leading to e.g., severe behavioral and personality changes, increased excitability, loss of memory, and insomnia^3^. Hence, many environmental and health-related departments, such as the United States Environmental Protection Agency (US EPA) and the World Health Organization (WHO), have designated mercury one of the most dangerous metal species^4^. Therefore, the development of a fast and convenient method for mercury determination has become an important issue in analytical and environmental sciences.

Several quantitative Hg^2+^ determination methods based on analytical instrumental techniques, such as atomic absorption spectroscopy (AAS)^5^, X-ray fluorescence spectroscopy (XRF)^6^, differential pulse anodic stripping voltammetry (DPASV)^7^, and capillary electrophoresis (CE)^8^ have been reported. Nevertheless, due to their high costs and technical difficulties in operation, the development of selective and sensitive signaling methods employing simple colorimetric or fluorescence changes is highly desirable. In this context, many UV–vis and/or fluorescence signaling methods using reaction-based probes have been developed^9–14^.

In particular, a large number of elaborately designed probes containing sulfur-based moieties have been designed to exploit the highly thiophilic nature of mercury in the determination of Hg^2+^ ions. Following the pioneering research of Czarnik’s anthracene thioamide-based Hg^2+^ signaling system^15^, many Hg^2+^-selective signaling probes using Hg^2+^-induced desulfurization of sulfur-based compounds, such as thiocarbonyl^16,17^, thioamide^18^, thioimide^19,20^, and thiourea derivatives^21–23^, have been reported. Furthermore, a number of probes exist which use the Hg^2+^-induced desulfurization-based cyclization of thioureas^24–26^, and cleavage of thioacetals^27–29^, thioethers^30^, thiophosphinates^31^, and thionocarbonates^32^. Sulfur-based functional groups in many of these probes effectively suppress the fluorescence of signaling subunits, and desulfurization results in the restoration of the absorption and fluorescence properties of the parent dyes^33^.

Following the introduction of microfluidic paper-based analytical devices (*μ*PADs)^34^, many paper-based kits for the determination of biologically and environmentally important materials have been reported. Paper-based devices manifest many features such as desirable mechanical properties (flexibility, stiffness, lightness, and thickness), economic feasibility, high surface-to-volume ratio, and capillary action^35–37^. In particular, using fabrication techniques such as drawing, stamping, cutting, and photolithography^38^, a number of *μ*PADs have been developed for the determination of important species including nitrites^39^, Hg^2+ ^^40^, Cu^2+ ^^41^, and Fe^3+ ^^42^. However, some of these methods suffer from high instrument costs and technical difficulties, and mass production is difficult in some cases. To overcome the drawbacks of *μ*PAD fabrication methods, the wax printing fabrication technique has been developed^43,44^, and successfully used for the preparation of *μ*PADs for the determination of biologically and environmentally important species including Cu^2+ ^^45^, Ni^2+ ^^46^, Hg^2+ ^^47^, Ca^2+^/Mg^2+ ^^48^, and organophosphate pesticides^49^.

Herein, we report a new resorufin thionocarbonate-based Hg^2+^-selective signaling probe and its application to *μ*PADs. The **RT** probe demonstrated a prominent color change from yellow to pink as well as remarkable turn-on type fluorescence signaling behavior for the Hg^2+^ ion. We confirmed that the Hg^2+^ signaling of **RT** was due to Hg^2+^-induced hydrolysis of the thionocarbonate moiety to form the parent resorufin dye, using ^1^H NMR and mass spectrometry. Furthermore, use of an **RT**-equipped *μ*PAD prepared using a wax printing technique enabled the simple and convenient determination of Hg^2+^ ions in simulated wastewater samples, with a detection limit of 5.9 × 10^−6^ M.

## Results and Discussion

### Preparation of RT

**Figure 1:**
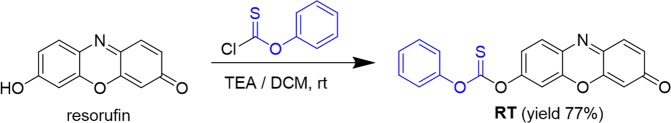
Preparation of the resorufin thionocarbonate derivative (**RT**).

The designed probe, **RT**, was synthesized by simple reaction of resorufin, which acts as a reporting chromophore as well as fluorophore^50^, with phenyl thionochloroformate (triethylamine, dichloromethane, yield = 77%) (Fig. 1)^32^. The chemical structure of **RT** was characterized by ^1^H NMR and ^13^C NMR measurements, and high-resolution mass spectrometry. As expected, due to the protection of the phenolic moiety of resorufin by thionocarbonate^51,52^, the probe exhibited a yellow color and weak fluorescence emission in a 1:1 (*v/v*) mixture of acetonitrile and citrate buffer solution (pH 6.2, final concentration = 10 mM).

### Hg^2+^-selective UV–vis and fluorescence signaling of RT

**Figure 2:**
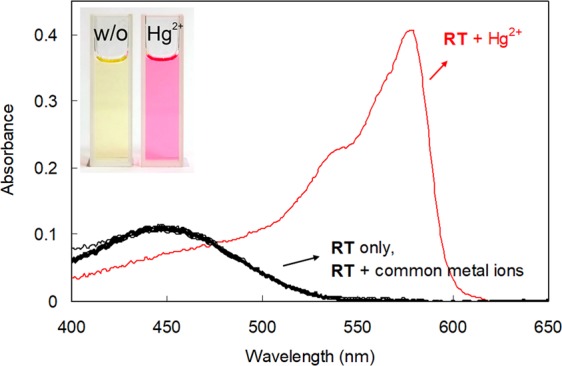
UV−vis spectra of **RT** in the presence and absence of various metal ions. [**RT**] = 1.0 × 10^−5^ M, [M^n+^] = 1.0 × 10^−4^ M in a 1:1 (*v/v*) mixture of citrate buffer solution (pH 6.2, 20 mM) and acetonitrile.

First, we investigated the UV–vis signaling behavior of **RT** in the presence of representative metal ions. In an acetonitrile/citrate buffer solution (1:1, pH 6.2), **RT** exhibited a broad absorption band centered around 448 nm (Fig. 2). The treatment of **RT** with common metal ions did not result in any significant changes in the absorption profile, except in the case of Hg^2+^ ions. In the presence of Hg^2+^, **RT** showed a strong absorption band at 578 nm and a prominent color change from yellow to pink. Based on this observation, the Hg^2+^ signaling of **RT** was assessed using the absorbance changes at 578 nm (Fig. S1, Supplementary Information). Absorbance enhancements (*A*/*A*_0_) at 578 nm clearly showed the prominent Hg^2+^-selective signaling behavior (*A*/*A*_0_ at 578 nm = 320 for **RT** in the presence of Hg^2+^), while the response to other metal ions was negligible (*A*/*A*_0_ varied between 0.1 for K^+^ and 1.4 for Ag^+^).

**Figure 3:**
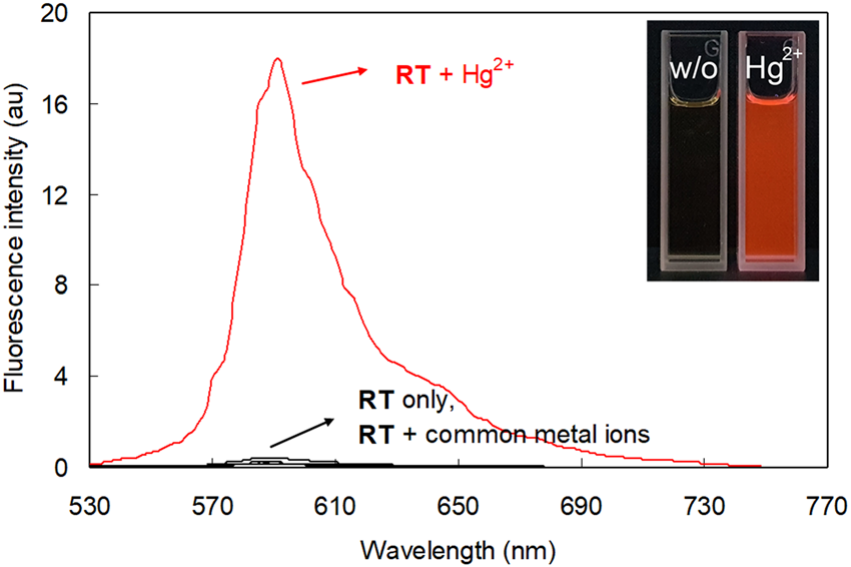
Fluorescence spectra of **RT** in the presence and absence of various metal ions. [**RT**] = 5.0 × 10^−6^ M, [M^n+^] = 5.0 × 10^−5^ M in a 1:1 (*v/v*) mixture of citrate buffer solution (pH 6.2, 20 mM) and acetonitrile. *λ*_ex_ = 478 nm.

Next, the fluorescence signaling behavior of **RT** in the presence of environmentally relevant metal ions was measured (Fig. 3). The **RT** showed a weak fluorescence emission centered around 587 nm due to the protection of the resorufin moiety by the phenyl thionocarbonate functionality. However, the treatment of **RT** with Hg^2+^ ions resulted in a prominent fluorescence enhancement at 591 nm (*I*/*I*_0_ at 591 nm = 101 for Hg^2+^). Other tested metal ions caused minor changes in fluorescence emission (i.e., *I*/*I*_0_ at 591 nm varied between 0.92 for Na^+^ and 2.29 for Ag^+^) (Fig. S2, Supplementary Information). These observations imply that the designed probe, **RT**, exhibited prominent, selective colorimetric and fluorescence signaling behavior toward Hg^2+^ ions. However, as this research is aimed at the development of paper-based Hg^2+^ determination tools, we focused on the colorimetric signaling behavior, which is more convenient for application in paper-based devices for use in the field than fluorescence signaling.

### Determination of Hg^2+^ by the RT probe

To detect Hg^2+^ ions in environmental and industrial samples, the selective signaling response to Hg^2+^ ions in the presence of relevant metal ions is a requisite. The Hg^2+^ signaling of **RT** was not affected by the presence of environmentally relevant metal ions (Fig. S3, Supplementary Information). The ratio of absorbance changes (*A*_Metal+Hg(II)_/*A*_Hg(II)_) at 578 nm only varied in a narrow range between 0.84 (Fe^3+^) and 1.0 (Li^+^). The pH-dependency of Hg^2+^ signaling by **RT** showed that signaling became more pronounced as the solution pH increased, up to pH 6.2, and was subsequently not influenced significantly by pH variation between pH 6.2 and 9.4 (Fig. S4, Supplementary Information). Therefore, signaling experiments were performed in citrate buffer solutions at pH 6.2, where the most prominent Hg^2+^ signaling contrast was observed. The Hg^2+^ signaling of **RT** was fast and completed within 5 min. From the signaling time course, we estimated that the rate constant of Hg^2+^ signaling under *pseudo-*first order conditions was 0.874 min^−1^ (Figs S5 and S6, Supplementary Information).

**Figure 4:**
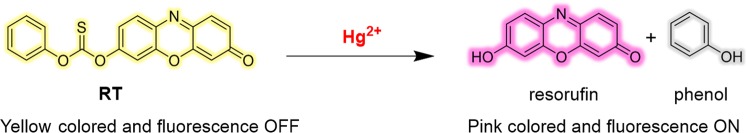
Hg^2+^ signaling process of **RT**.

**Figure 5:**
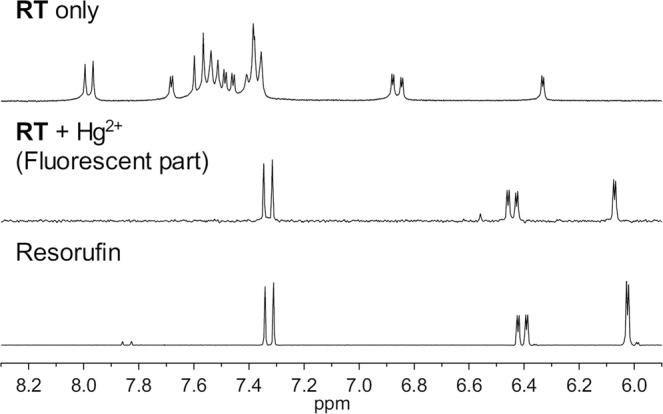
Partial ^1^H NMR spectra of **RT**, **RT** with Hg^2+^ ions (fluorescent part), and resorufin. [**RT**] = [resorufin] = 10 mM in DMSO-*d*_6_. NMR spectrum of **RT** with Hg^2+^ (**RT** + Hg^2+^), fluorescent part) was obtained after simple purification of the signaling product using a short silica column.

The Hg^2+^ signaling of **RT** was due to the Hg^2+^-induced cleavage reaction of thionocarbonate moiety of **RT** to yield the parent resorufin dye and non-fluorescent phenol as a side product (Fig. 4). The postulated Hg^2+^ signaling process of **RT** was confirmed by ^1^H NMR and mass measurements. As shown in Fig. 5, we found that the ^1^H NMR spectrum of the signaling product (**RT** + Hg^2+^) was nearly identical to that of the postulated signaling product resorufin. Furthermore, the mass spectrum of the signaling product of **RT** with the Hg^2+^ ion revealed a diagnostic peak at *m*/*z* = 213, which is consistent with the proposed signaling product, resorufin (calcd. for [C_12_H_7_NO_3_]^+^, *m*/*z* = 213.0) (Fig. S7, Supplementary Information). We also characterized the signaling side product, phenol, by ^1^H NMR and ^13^C NMR measurements (Figs S8 and S9, Supplementary Information).

**Figure 6:**
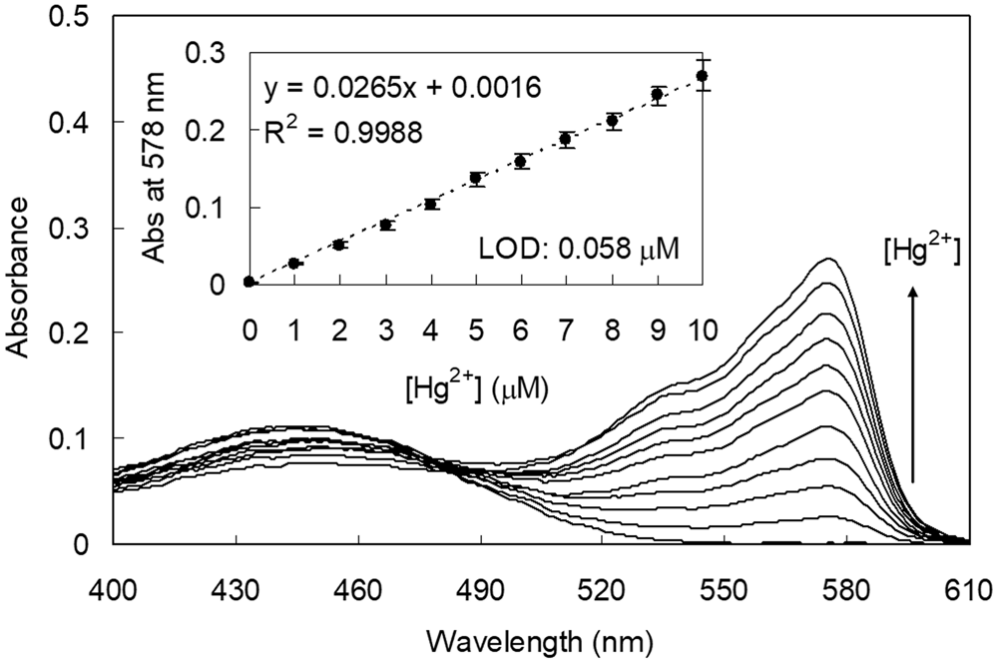
UV–vis titration of **RT** with Hg^2+^. Inset: changes in absorbance of **RT** at 578 nm as a function of [Hg^2+^]. [**RT**] = 1.0 × 10^−5^ M, [Hg^2+^] = 0–1.0 × 10^−5^ M in a 1:1 (*v/v*) mixture of citrate buffered simulated wastewater (pH 6.2, 20 mM) and acetonitrile.

Next, to estimate the detection limit of **RT** for the Hg^2+^ ion, the Hg^2+^ concentration-dependent UV–vis absorbance change was measured. As shown in Fig. 6, the absorbance at 578 nm increased linearly with the increase of Hg^2+^, up to 1.0 × 10^−5^ M. From this measurement, the detection limit of **RT** for Hg^2+^ ion was calculated to be 6.3 × 10^−8^ M, according to the IUPAC guideline (3*s*_bl_/*m*)^53^. In addition, because the determination of mercury pollution is one of the most critical challenges in environmental and industrial fields, we attempted to determine Hg^2+^ in simulated wastewater samples^54^. The titration of **RT** with Hg^2+^ showed a satisfactory calibration plot up to 1.0 × 10^−5^ M. From these concentration-dependent experiments, the detection limit for Hg^2+^ in simulated wastewater was estimated to be 5.8 × 10^−8^ M (Fig. S10, Supplementary Information).

### Development of the RT-based µPAD

**Figure 7:**
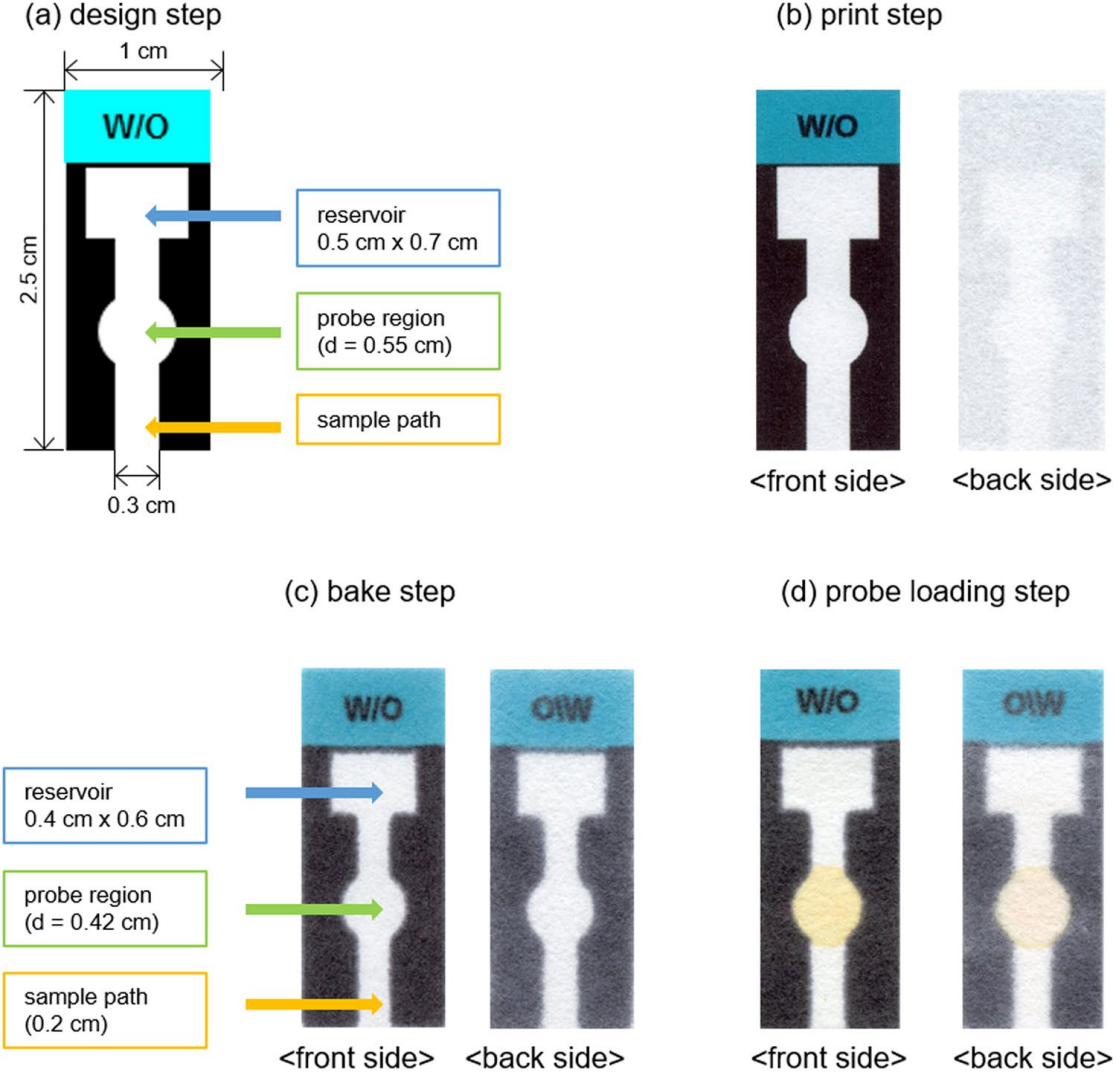
Preparation of **RT**-equipped *μ*PAD.

**Figure 8:**
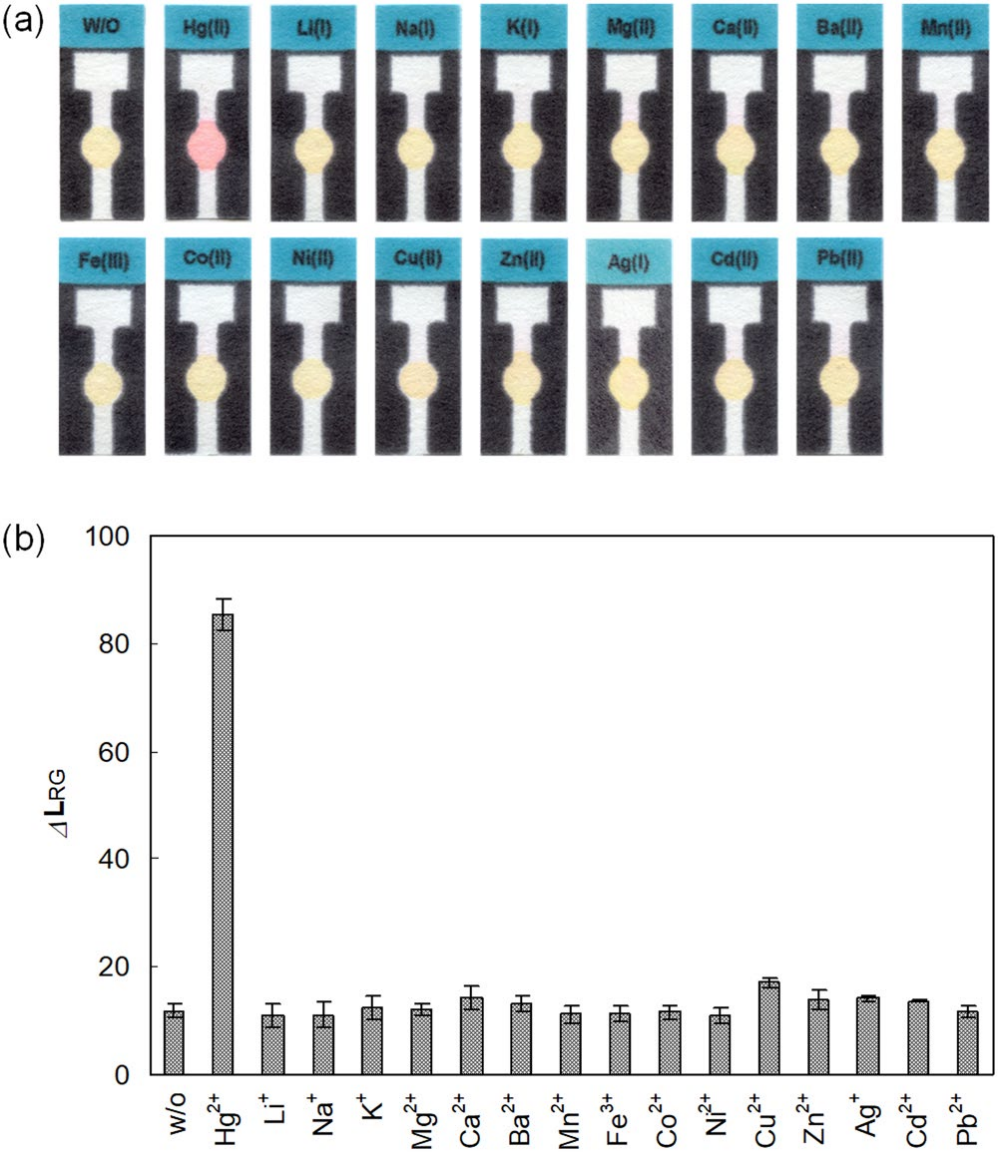
(**a**) Photograph and (**b**) RGB analysis (assessed as (*Δ***L**_RG_** = L**_red_ − **L**_green_) of color changes of the **RT**-equipped *μ*PAD kit in the presence and absence of representative metal ions. [M^n+^] = 5.0 × 10^−4^ M in tris buffer solution (pH 8.0, final concentration = 10 mM). The reaction time was 10 min, which is the sum of the time required for the full elution of the analyte to the top of the reservoir and subsequent drying. Due to the formation of the insoluble AgCl precipitate in tris-HCl buffer solution, the Ag^+^ result was obtained after filtration of the turbid solution using a syringe filter (0.2 μm). The error bars were obtained from three independent experiments.

Recently, due to their convenience and cost-effectiveness, many probes constructed on *μ*PADs have been developed for the determination of a variety of biological and environmental species^35–37^. Herein, we utilized the newly developed Hg^2+^ probe as a *μ*PAD-based colorimetric signaling tool to increase its convenience in practical applications by eliminating the need for complicated instruments operated by well-trained professionals^43^. The designed *μ*PAD tool was easily prepared by wax printing, based on the design shown in Fig. 7. First, using the *μ*PAD kit, we investigated the effect of pH on the Hg^2+^ signaling behavior in various buffer solutions. As shown in Fig. S11 (Supplementary Information), the paper kit exhibited the most prominent color change in response to Hg^2+^ ions in tris-HCl buffer solution at pH 8.0. Next, the colorimetric signaling behavior of the *μ*PAD kit in the presence of representative metal ions was studied. An **RT**-equipped *μ*PAD kit is light-yellow in color, and no significant color change was observed in the presence of common metal ions except in the case of Hg^2+^ ions (Fig. 8(a)). Upon treatment with Hg^2+^ ions, the *μ*PAD kit underwent a pronounced color change from yellow to pink. Subsequently, we investigated the Hg^2+^ signaling behavior of the paper kit by RGB color analysis. As shown in Fig. 8(b), the *Δ***L**_RG_ value, assessed by the difference between the red and green channel levels (**L**_red_ − **L**_green_), clearly revealed the Hg^2+^-selective signaling behavior. For instance, the control *Δ***L**_RG_ value of the *μ*PAD kit was 11.83 for distilled water, whereas the value increased to 85.47 on exposure to Hg^2+^ ions. In the presence of thiophilic Ag^+^, noticeable interference was observed due to the reactivity of the thionocarbonate moiety of probe **RT** with Ag^+^ ions. However, interference from Ag^+^ was dramatically suppressed by use of a tris-HCl buffer as an Ag^+^ masking agent, due to the formation of an insoluble AgCl precipitate (*K*_sp_ = 1.8 × 10^−10^) by interaction of Ag^+^ with chloride ions in the tris-HCl buffer solution (*Δ***L**_RG_ = 21.35 for Ag^+^). However, considerable interference was still observed, as the residual AgCl in the measurement solutions released a small amount of Ag^+^ which induced a considerable colorimetric response in the *μ*PAD. We believed that Ag^+^ ions released from AgCl subsequently react with **RT** to form much more insoluble Ag_2_S (*K*_sp_ of Ag_2_S = 8.0 × 10^−51^) which induced a colorimetric response in the **RT**-equipped *μ*PAD. Thus, we removed the residual AgCl precipitate using a syringe filter and found that the interference from Ag^+^ decreased significantly (*Δ***L**_RG_ = 13.12 for Ag^+^, filtered) (Fig. S12, Supplementary Information). Furthermore, we confirmed that the suppression of Ag^+^ interference on the **RT**-equipped *μ*PAD by syringe filtration was maintained for at least 30 min during the signaling experiment (Fig. S13, Supplementary Information). We also confirmed that the Hg^2+^ signaling by the **RT**-equipped *μ*PAD kit was not affected by the filtration process (Fig. S14, Supplementary Information). Other metal ions showed insignificant changes (*Δ***L**_RG_ values ranged from 10.99 for Li^+^ to 17.55 for Cu^2+^). Finally, the Hg^2+^ signaling by the *μ*PAD was evaluated quantitatively, by plotting *Δ***L**_RG_ values as a function of [Hg^2+^] (Figs S15 and S16, Supplementary Information). The *Δ***L**_RG_ value steadily increased with an increase in Hg^2+^ concentration, exhibiting a linear relationship up to 2.0 × 10^−4^ M Hg^2+^ ions. In fact, it might be possible to obtain a distorted Hg^2+^ titration plot in the presence of a low concentration of Hg^2+^ as the Hg^2+^ interacts first with the bottom part of the spotted probe. However, a satisfactory calibration curve with a good linear relationship (*R*^2^ = 0.9945) for Hg^2+^ in the concentration range between 0 and 50 μM was obtained (Fig. S16, Supplementary Information). That might be because the time required for the expression of a stable Hg^2+^-induced response from the probe was approximately 5 min (Fig. S5, Supplementary Information), while that for the full elution of the analyte up to the top of the *μ*PAD reservoir was approximately 1 min. Thus Hg^2+^ ions are eluted quite evenly over the entire spotted probe area before the chromogenic signaling occurs. From the Hg^2+^ concentration-dependent signaling profile, the detection limit for Hg^2+^ ions by the paper kit was estimated as 4.5 × 10^−6^ M, according to IUPAC guidelines (Fig. S16, Supplementary Information). Based on these results, to demonstrate practical application, the determination of Hg^2+^ in a simulated wastewater sample was conducted using an **RT**-equipped *μ*PAD kit, and the detection limit for Hg^2+^ in simulated wastewater was calculated to be 5.9 × 10^−6^ M (Fig. S17, Supplementary Information). In fact, the detection limit of **RT**-equipped *μ*PAD for the Hg^2+^ ion is not superior to those of previously reported *μ*PAD systems^40,47^. However, the **RT**-equipped *μ*PAD is more easily prepared and can determine the mercury ions by a simple elution procedure compared with previously reported *μ*PAD systems, which required extra treatment of the analytes.

## Conclusion

We have developed the new resorufin thionocarbonate-based Hg^2+^ signaling probe **RT** which operates via the selective hydrolysis of thionocarbonate with Hg^2+^ ions. **RT** showed a significant color change from yellow to pink as well as prominent fluorogenic signaling behavior exclusively toward Hg^2+^ ions. Selective Hg^2+^- signaling by **RT** was unaffected by the presence of common metal ions as a background, and the detection limit of Hg^2+^ in a simulated wastewater sample was calculated to be 5.8 × 10^−8^ M. From the ^1^H NMR, ^13^C NMR, and mass spectral measurements, we confirmed that the Hg^2+^ signaling of **RT** was due to Hg^2+^-induced hydrolysis of the phenyl thionocarbonate moiety of **RT** into its parent resorufin dye and phenol. To demonstrate the practical application of the probe, the determination of Hg^2+^ ion using an **RT**-equipped *μ*PAD was performed. With the **RT**-equipped *μ*PAD, the convenient determination of Hg^2+^ ions in simulated wastewater was realized with a detection limit of 5.9 × 10^−6^ M, using a readily available office scanner as an image capturing tool.

## Methods

### General

Resorufin, phenyl thionochloroformate, and triethylamine were purchased from Aldrich Chemical Co. All solvents were of spectroscopic grade and obtained from Aldrich Chemical Co. ^1^H NMR and ^13^C NMR spectra were acquired on a Varian VNS NMR spectrometer (^1^H NMR: 600 MHz, ^13^C NMR 150 MHz). UV–vis and fluorescence spectra were measured using a Scinco S-3100 spectrophotometer and a Scinco FS-2 fluorescence spectrophotometer, respectively. High-resolution mass spectrometry (HRMS) results were obtained on a JEOL JMS-700 mass spectrometer using fast atom bombardment (FAB) ionization. Column chromatography was performed using silica gel (Merck, 240 mesh).

### Preparation of Hg^2+^ signaling probe, RT

Resorufin (0.21 g, 1.0 mmol) was dissolved in dichloromethane (30 mL), and triethylamine (0.27 mL, 2.0 mmol) was added to the solution. After 30 min of stirring, phenyl thionochloroformate (0.21 mL, 1.5 mmol) was added dropwise, and the reaction mixture was stirred at room temperature for 12 h. The reaction mixture was washed with distilled water and brine several times. The solution was evaporated under reduced pressure and the remaining residue was purified by column chromatography (silica gel, eluent: dichloromethane) to yield **RT** (0.27 g, 77%) as a scarlet powder. ^1^H NMR (600 MHz, CDCl_3_): δ 7.87 (d, *J* = 8.4 Hz, 1 H), 7.48 (dd, *J* = 8.6, 7.3 Hz, 2 H), 7.44 (d, *J* = 9.8 Hz, 1 H), 7.35 (t, *J* = 7.4 Hz, 1 H), 7.27–7.20 (m, 4 H), 6.87 (dd, *J* = 9.8, 2.0 Hz, 1 H), 6.34 (d, *J* = 2.0 Hz, 1 H); ^13^C NMR (150 MHz, CDCl_3_): δ 193.5, 186.2, 155.4, 153.4, 149.1, 148.9, 144.4, 135.4, 134.8, 131.9, 131.4, 129.8, 127.1, 121.6, 119.7, 110.5, 107.4; HRMS (FAB^+^, *m/z*): calcd for C_19_H_12_NO_4_S^+^ [M + H]^+^: 350.0482, found 350.0483.

### Preparation of stock solutions

A stock solution of **RT** (5.0 × 10^−4^ M) was prepared in spectroscopic grade acetonitrile. Stock solutions of the metal ions (10 mM) were prepared by dissolving the metal perchlorate salts in distilled water. ***Caution***: metal perchlorates are highly explosive, thus should be handled carefully and used in small quantities. The simulated wastewater was prepared by following the reported literature procedure^54^. Composition of the simulated wastewater: [NaClO_4_] = 2.39 mM, [NaHCO_3_] = 1.23 mM, [NaCl] = 987 μM, [NaNO_3_] = 484 μM, [Mg(ClO_4_)_2_] = 288 μM, [KClO_4_] = 281 μM, [Ca(ClO_4_)_2_] = 250 μM, [Na_2_SO_4_] = 208 μM, [NaH_2_PO_4_] = 105 μM, [NaF] = 16 μM.

### Evidence of the Hg^2+^ signaling process of RT

A solution of **RT** (0.035 g, 0.01 mmol) in 10 mL of methanol was slowly added to mercury perchlorate (0.12 g, 0.03 mmol) in methanol. The signaling progress was monitored by TLC. Due to the low solubility of resorufin in methanol, a simple filtration was sufficient for the separation of the primary signaling product resorufin and side product phenol in the filtrate. ^1^H NMR and mass spectral measurements of the solid product (resorufin) were performed after column chromatography. The ^1^H NMR and ^13^C NMR of phenol were obtained after the evaporation of the filtrate.

### Determination of Hg^2+^ using the *μ*PAD

The *μ*PAD was designed using Microsoft Office Power Point. The *μ*PAD was printed on Whatman® cellulose chromatography paper using a commercial wax printer (Fuji Xerox, ColorQube 8570)^43^. After printing, the paper was baked in a 150 °C oven for 90 s and cut into separate *μ*PADs. The **RT** was dissolved in dichloromethane (8.0 mM) and loaded onto the middle of the circle in the prepared *μ*PAD using a capillary tube (Aldrich, Z114952) (applied volume = 1.6 μL). Varying concentrations of Hg^2+^ in simulated wastewater sample were added to the **RT**-equipped *μ*PAD kit, which resulted in a color change from yellow to pink. The analyte solution was prepared by adding 1% (*v/v*) of tris buffer solution (pH 8.0, 1.0 M) to the wastewater stock solution. Synthetic wastewater samples with varying concentrations of Hg^2+^ were prepared, and each sample was added to a separate vial. The bottom of an **RT**-equipped *μ*PAD kit was placed in each vial and the analyte solution was eluted to the top of the *μ*PAD. The *μ*PAD was removed and dried under ambient conditions for 10 min. The results were recorded using a flatbed scanner (Epson, Perfection V550 Photo Color Scanner), and part of the **RT** spot on the *μ*PAD, encircling 4.0 mm from the center, was analyzed using the Photoshop program (Adobe, Photoshop CS6). We chose to analyze a subsection of the spot due to the change in the size of the spot to approximately 4.2–4.3 mm in diameter, caused by broadening of the wax barrier during the baking step, and to the absence of a clear boundary line between the **RT** spot and the wax barrier of the *μ*PAD.

## Supplementary information

Supplementary Information

## References

[1] Simon, M., Jonk, P., Wuhl-Couturier, G. & Halbach, S. Ullmann’s Encyclopedia of Industrial Chemistry Mercury, Mercury Alloys, and Mercury Compounds (Wiley-VCH Verlag GmbH & Co. KGaA, 2012).

[2] Levin M, Jacobs J, Polos PG (1988). Acute Mercury Poisoning and Mercurial Pneumonitis from Gold Ore Purification. Chest.

[3] Bidstrup PL, Bonnel JA, Harvey DG, Locket S (1951). Chronic Mercury Poisoning in Men Repairing Direct-Current Meters. Lancet.

[4] Guidelines for Drinking-water Quality (World Health Organization, 1996).

[5] Carnrick GR, Barnett W, Slavin W (1986). Spectral Interferences Using the Zeeman Effect for Furnace Atomic Absorption Spectroscopy. Spectrochim. Acta Part B.

[6] Dumarey R, Heindryckx R, Dams R, Hoste J (1979). Determination of Volatile Mercury Compounds in Air with the Coleman Mercury Analyzer System. Anal. Chim. Acta.

[7] Bloom, H. & Noller, B. Trends in Electrochemistry 241–252 (Badford, 1976).

[8] Medina I, Rubí E, Mejuto MC, Cela R (1993). Speciation of Organomercurials in Marine Samples using Capillary Electrophoresis. Talanta.

[9] Nolan EM, Lippard SJ (2008). Tools and Tactics for the Optical Detection of Mercuric Ion. Chem. Rev..

[10] Li X, Gao X, Shi W, Ma H (2014). Design Strategies for Water-Soluble Small Molecular Chromogenic and Fluorogenic Probes. Chem. Rev..

[11] Carter KP, Young AM, Palmer AE (2014). Fluorescent Sensors for Measuring Metal Ions in Living Systems. Chem. Rev..

[12] Yang Y, Zhao Q, Feng W, Li F (2013). Luminescent Chemodosimeters for Bioimaging. Chem. Rev..

[13] Du J, Hu M, Fan J, Peng X (2012). Fluorescent Chemodosimeters Using “Mild” Chemical Events for the Detection of Small Anions and Cations in Biological and Environmental Media. Chem. Soc. Rev..

[14] Yuan L, Lin W, Zheng K, He L, Huang W (2013). Far-Red to Near Infrared Analyte-Responsive Fluorescent Probes based on Organic Fluorophore Platforms for Fluorescence Imaging. Chem. Soc. Rev..

[15] Chae M-Y, Czarnik AW (1992). Fluorometric Chemodosimetry. Mercury(II) and Silver(I) Indication in Water via Enhanced Fluorescence Signaling. J. Am. Chem. Soc..

[16] Zhang, G., *et al* 1,3-Dithiole-2-thione Derivatives Featuring an Anthracene Unit: New Selective Chemodosimeters for Hg(II) Ion. *Chem. Commun*. 2161–2163 (2005). 10.1039/b417952h 15846433

[17] Choi, M. G., Kim, Y. H., Namgoong, J. E. & Chang, S.-K. Hg^2+^-Selective Chromogenic and Fluorogenic Chemodosimeter Based on Thiocoumarins. *Chem. Commun*. 3560–3562 (2009). 10.1039/b905612b 19521607

[18] Song KC (2006). Fluorogenic Hg^2+^-Selective Chemodosimeter Derived from 8-Hydroxyquinoline. Org. Lett..

[19] Moon JO, Choi MG, Sun T, Choe J-I, Chang S-K (2013). Synthesis of Thionaphthalimides and Their Dual Hg^2+^-Selective Signaling by Desulfurization of Thioimides. Dyes Pigment..

[20] Li M, Li X-J, Lu H-Y, Chen C-F (2014). Tetrahydro[5]helicene Thioimide-Based Fluorescent and Chromogenic Chemodosimeter for Highly Selective and Sensitive Detection of Hg^2+^. Sens. Actuator B-Chem..

[21] Tsukamoto K, Shinohara Y, Iwasaki S, Maeda H (2011). A Coumarin-Based Fluorescent Probe for Hg^2+^ and Ag^+^ with an *N*’-Acetylthioureido Group as a Fluorescence Switch. Chem. Commun..

[22] Yan Y, Zhang Y, Xu H (2013). A Selective “Turn‐On” Fluorescent Probe for Recognition of Mercury(II) Ions in Aqueous Solution Based on a Desulfurization Reaction. ChemPlusChem.

[23] Areti S (2014). Glyco-Conjugate as Selective Switch-on Molecule for Hg^2+^ in the Presence of Albumin Proteins, Blood Serum Milieu and on Silica Gel Solid Support. RSC Adv..

[24] Yang Y-K, Yook K-J, Tae J (2005). A Rhodamine-Based Fluorescent and Colorimetric Chemodosimeter for the Rapid Detection of Hg^2+^ Ions in Aqueous Media. J. Am. Chem. Soc..

[25] Zou Q, Tian H (2010). Chemodosimeters for Mercury(II) and Methylmercury(I) Based on 2,1,3-Benzothiadiazole. Sens. Actuator B-Chem..

[26] Srivastava P, Ali R, Razi SS, Shahid M, Misra A (2013). Thiourea Based Molecular Dyad (ANTU): Fluorogenic Hg^2+^ Selective Chemodosimeter Exhibiting Blue–Green Fluorescence in Aqueous-Ethanol Environment. Sens. Actuator B-Chem..

[27] Rao AS (2012). Reaction-Based Two-Photon Probes for Mercury Ions: Fluorescence Imaging with Dual Optical Windows. Org. Lett..

[28] Dai H (2012). A Selective and Sensitive “Turn‐on” Fluorescent Chemosensor for Recognition of Hg^2+^ Ions in Water. Chem. Eur. J..

[29] Song C (2015). Fluorescent Theranostic Agents for Hg^2+^ Detection and Detoxification Treatment. Chem. Commun..

[30] Kim D, Yamamoto K, Ahn KH (2012). BODIPY-Based Reactive Probe for Ratiometric Fluorescence Sensing of Mercury Ions. Tetrahedron.

[31] Im HG, Kim HY, Chang S-K (2014). Dual Signaling of Hg^2+^ Ions by Selective Cleavage of Thiophosphinated Rhodol. Sens. Actuator B-Chem..

[32] Shu W (2016). Novel Carbonothioate-Based Colorimetric and Fluorescent Probe for Selective Detection of Mercury Ions. Ind. Eng. Chem. Res..

[33] Goldberg JM, Batjargal S, Chen BS, Petersson EJ (2013). Thioamide Quenching of Fluorescent Probes through Photoinduced Electron Transfer: Mechanistic Studies and Applications. J. Am. Chem. Soc..

[34] Martinez AW, Phillips ST, Butte MJ, Whitesides GM (2007). Patterned Paper as a Platform for Inexpensive, Low‐Volume, Portable Bioassays. Angew. Chem. Int. Ed..

[35] Almeida MIGS, Jayawardane BM, Koleva SD, McKelvie ID (2018). Developments of Microfluidic Paper-Based Analytical Devices (μPADs) for Water Analysis: A Review. Talanta.

[36] Yang Y (2017). Paper-Based Microfluidic Devices: Emerging Themes and Applications. Anal. Chem..

[37] Nery EW, Kubota LT (2013). Sensing Approaches on Paper-Based Devices: A Review. Anal. Bioanal. Chem..

[38] Cate DM, Adkins JA, Mettakoonpitak J, Henry CS (2015). Recent Developments in Paper-Based Microfluidic Devices. Anal. Chem..

[39] Cardoso TMG, Garcia PT, Coltro WKT (2015). Colorimetric Determination of Nitrite in Clinical, Food and Environmental Samples using Microfluidic Devices Stamped in Paper Platforms. Anal. Methods.

[40] Ruan Z, Li C, Rong J-R, Qin J, Li Z (2015). A Relay Strategy for the Mercury (II) Chemodosimeter with Ultra-Sensitivity as Test Strips. Sci. Rep..

[41] Ratnarathorn N, Chailapakul O, Henry CS, Dungchai W (2012). Simple Silver Nanoparticle Colorimetric Sensing for Copper by Paper-Based Devices. Talanta.

[42] Asano H, Shiraishi Y (2015). Development of Paper-Based Microfluidic Analytical Device for Iron Assay using Photomask Printed with 3D Printer for Fabrication of Hydrophilic and Hydrophobic Zones on Paper by Photolithography. Anal. Chim. Acta.

[43] Carrilho E, Martinez AW, Whitesides GM (2009). Understanding Wax Printing: A Simple Micropatterning Process for Paper-Based Microfluidics. Anal. Chem..

[44] Lu Y, Shi W, Jiang L, Qin J, Lin B (2009). Rapid Prototyping of Paper-Based Microfluidics with Wax for Low-Cost, Portable Bioassay. Electrophoresis.

[45] Pratiwi R (2017). A Selective Distance-Based Paper Analytical Device for Copper(II) Determination using a Porphyrin Derivative. Talanta.

[46] Ota R, Yamada K, Suzuki K, Citterio D (2018). Quantitative Evaluation of Analyte Transport on Microfluidic Paper-Based Analytical Devices (μPADs). Analyst.

[47] Chen G-H (2014). Detection of Mercury(II) Ions Using Colorimetric Gold Nanoparticles on Paper-Based Analytical Devices. Anal. Chem..

[48] Karita S, Kaneta T (2016). Chelate Titrations of Ca^2+^ and Mg^2+^ using Microfluidic Paper-Based Analytical Devices. Anal. Chim. Acta.

[49] Sicard C (2015). Tools for Water Quality Monitoring and Mapping using Paper-Based Sensors and Cell Phones. Water Res..

[50] Bueno C (2002). The Excited-State Interaction of Resazurin and Resorufin with Amines in Aqueous Solutions. Photophysics and Photochemical Reactions. Photochem. Photobiol..

[51] Choi MG, Hwang JY, Eor SY, Chang S-K (2010). Chromogenic and Fluorogenic Signaling of Sulfite by Selective Deprotection of Resorufin Levulinate. Org. Lett..

[52] Choi MG, Moon JO, Bae J, Lee JW, Chang S-K (2013). Dual Signaling of Hydrazine by Selective Deprotection of Dichlorofluorescein and Resorufin acetates. Org. Biomol. Chem..

[53] Harris, D. C. Quantitative Chemical Analysis (8th ed.) 103–105 (W. H. Freeman and Company, 2010).

[54] Tchobanoglous, G. & Burton, F. L. Wastewater engineering: treatment disposal reuse 1820 (McGraw-Hill, 1991).

