# Supplementary material for: Novel Hg2+-Selective Signaling Probe Based on Resorufin Thionocarbonate and its μPAD Application
Source: Sci Rep. 2019 Mar 4;9:3348. doi: 10.1038/s41598-019-40169-6 (PMC6399246; doi:10.1038/s41598-019-40169-6)
Supplement: Supplementary file 1 — Supplementary Information [file 41598_2019_40169_MOESM1_ESM.docx]

**Supplementary Information**

**Novel Hg^2+^-Selective Signaling Probe Based on Resorufin Thionocarbonate and Its *μ*PAD Application**

*Myung Gil Choi, So Young Park, Ka Young Park, and Suk-Kyu Chang**

Department of Chemistry, Chung-Ang University, Seoul 06974, Republic of Korea

* To whom correspondence should be addressed:

Suk-Kyu Chang, Department of Chemistry, Chung-Ang University, Seoul 06974, Republic of Korea

Tel.: +82-2-820-5199; Fax: +82-2-825-4736; E­mail: skchang@cau.ac.kr

**Contents**

**Figure S1.** Changes in absorbance enhancement (*A*/*A*_0_) of **RT** at 578 nm in the presence and absence of various metal ions.

**Figure S2.** Changes in fluorescence emission of **RT** at 591 nm in the presence and absence of various metal ions.

**Figure S3.** Competitive signaling of Hg^2+^ ions by **RT** in the presence of representative metal ions as a background.

**Figure S4.** Effect of pH on the Hg^2+^ signaling by **RT**.

**Figure S5.** Time-dependent Hg^2+^ signaling of **RT** monitored at 578 nm.

**Figure S6.** *Pseudo*-first-order kinetic plot of the reaction of **RT** with Hg^2+^.

**Figure S7.** Mass spectrum of Hg^2+^-signaling product of **RT**.

**Figure S8.** Partial ^1^H NMR spectra of **RT** alone, **RT** with Hg^2+^ ions (non-fluorescent part), and phenol.

**Figure S9.** Partial ^13^C NMR spectra of **RT** alone, **RT** with Hg^2+^ ions (non-fluorescent part), and phenol.

**Figure S10.** Changes in absorbance of **RT** at 578 nm as a function of Hg^2+^ concentration in simulated wastewater sample.

**Figure S11.** Photograph and RGB analysis of color changes of **RT**-equipped *μ*PAD kit in varying pH conditions.

**Figure S12.** Suppression of the Ag^+^ interference of the **RT**-equipped *μ*PAD kit by chloride containing buffer and syringe filtration.

**Figure S13.** Time-dependent *Δ***L**_RG_ changes of **RT**-equipped *μ*PAD kit in the presence and absence of Ag^+^ ion.

**Figure S14.** Effect of filtration on the Hg^2+^ signaling by the **RT**-equipped *μ*PAD kit.

**Figure S15.** Changes in *Δ***L**_RG_ (**L**_red_ – **L**_green_) of **RT**-equipped *μ*PAD kit as a function of Hg^2+^ concentration ([Hg^2+^] = 0–4.0 × 10^–4^ M).

**Figure S16.** Changes in *Δ***L**_RG_ (**L**_red_ – **L**_green_) of **RT**-equipped *μ*PAD kit as a function of Hg^2+^ concentration ([Hg^2+^] = 0–5.0 × 10^–5^ M).

**Figure S17.** Changes in *Δ***L**_RG_ **(L**_red_ – **L**_green_) of **RT**-equipped *μ*PAD kit as a function of Hg^2+^ concentration in simulated wastewater sample.

**Figure S18.** ^1^H NMR spectrum of **RT** in CDCl_3_.

**Figure S19.** ^13^C NMR spectrum of **RT** in CDCl_3_.

**Figure S20.** High resolution FAB mass spectrum of **RT**.

**Figure S1.** Changes in absorbance enhancement (*A*/*A*_0_) of **RT** at 578 nm in the presence and absence of various metal ions. [**RT**] = 1.0 × 10^–5^ M, [M^n+^] = 1.0 × 10^–4^ M in a 1:1 (*v/v*) mixture of citrate buffer solution (pH 6.2, 20 mM) and acetonitrile.

**Figure S2.** Changes in fluorescence emission of **RT** at 591 nm in the presence and absence of various metal ions. [**RT**] = 5.0 × 10^–6^ M, [M^n+^] = 5.0 × 10^–5^ M in a 1:1 (*v/v*) mixture of citrate buffer solution (pH 6.2, 20 mM) and acetonitrile. λ_ex_ = 478 nm.

**Figure S3.** Competitive signaling of Hg^2+^ ions by **RT** in the presence of representative metal ions as a background. [**RT**] = 1.0 × 10^–5^ M, [Hg^2+^] = [M^n+^] = 1.0 × 10^–4^ M in a 1:1 (*v/v*) mixture of citrate buffer solution (pH 6.2, 20 mM) and acetonitrile.

**Figure S4.** Effect of pH on the Hg^2+^ signaling by **RT**. [**RT**] = 1.0 × 10^–5^ M, [Hg^2+^] = 1.0 × 10^–4^ M in a 1:1 (*v/v*) mixture of buffer solution (pH 3.0–6.2: citrate buffer, pH 7.0–9.4: citrate buffer adjusted with NaOH, final concentration = 20 mM) and acetonitrile.

**Figure S5.** Time-dependent Hg^2+^ signaling of **RT** monitored at 578 nm. [**RT**] = 1.0 × 10^–5^ M, [Hg^2+^] = 1.0 × 10^–4^ M in a 1:1 (*v/v*) mixture of citrate buffer solution (pH 6.2, 20 mM) and acetonitrile.

**Figure S6.** *Pseudo*-first-order kinetic plot of the reaction of **RT** with Hg^2+^. [**RT**] = 1.0 × 10^–5^ M, [Hg^2+^] = 1.0 × 10^–4^ M in a 1:1 (*v/v*) mixture of citrate buffer solution (pH 6.2, 20 mM) and acetonitrile. The rate constant of Hg^2+^ signaling was estimated under the *pseudo*-first order rate conditions using a large excess of Hg^2+^. The *pseudo*-first order rate constant was calculated following the reported literatures.^S1,S2^

**Figure S7.** Mass spectrum of Hg^2+^-signaling product of **RT**.

**Figure S8.** Partial ^1^H NMR spectra of **RT** alone, **RT** with Hg^2+^ ions (non-fluorescent part), and phenol. [**RT**] = [phenol] = 0.01 M in DMSO-*d*_6_. NMR spectrum of **RT** with Hg^2+^ (non-fluorescent part) was obtained after simple filtration and evaporation of a reaction mixture of **RT** with Hg^2+^ in methanol.

**Figure S9.** Partial ^13^C NMR spectra of **RT** alone, **RT** with Hg^2+^ ions (non-fluorescent part), and phenol. [**RT**] = [phenol] = 0.01 M in DMSO-*d*_6_. NMR spectrum of **RT** with Hg^2+^ (non-fluorescent part) was obtained after simple filtration and evaporation of a reaction mixture of **RT** with Hg^2+^ in methanol.

**Figure S10.** Changes in absorbance of **RT** at 578 nm as a function of Hg^2+^ concentration in simulated wastewater sample. [**RT**] = 1.0 × 10^–5^ M, [M^n+^] = 0–1.0 × 10^–5^ M in a 1:1 (*v/v*) mixture of citrate buffer solution (pH 6.2, 20 mM) and acetonitrile.


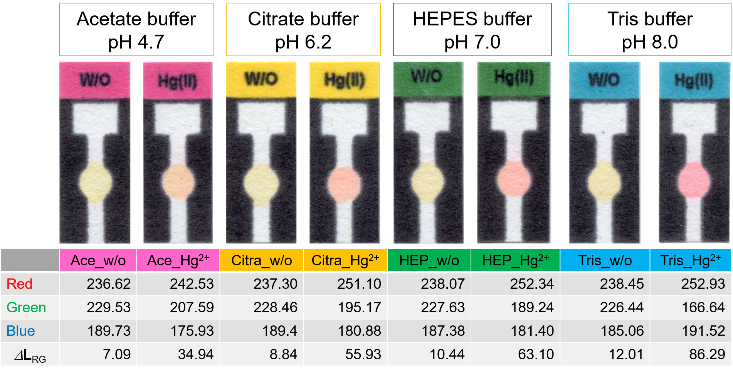


**Figure S11.** Photograph and RGB analysis of color changes of **RT**-equipped *μ*PAD kit in varying pH conditions. [Hg^2+^] = 5.0 × 10^–4^ M in a buffer solution (pH 4.7: acetate buffer, pH 6.2: citrate buffer, pH 7.0: HEPES buffer, pH 8.0: tris-HCl buffer, final concentration = 10 mM).


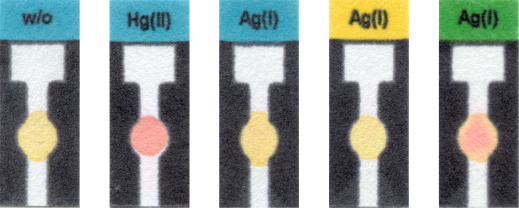


**Figure S12.** Suppression of the Ag^+^ interference of the **RT**-equipped *μ*PAD kit by chloride containing buffer and syringe filtration. [Hg^2+^] = [Ag^+^] = 5.0 × 10^–4^ M in tris-HCl buffer (pH = 8.0, final concentration = 10 mM). The result of Ag(I)-filtered was obtained after filtration of the solution using a syringe filter (0.2 μm). For comparison, result of Ag(I) containing sample obtained using a phosphate buffer solution (chloride free, pH = 8.0, final concentration = 10 mM) was shown.

**Figure S13.** Time-dependent *Δ***L**_RG_ changes of **RT**-equipped *μ*PAD kit in the presence and absence of Ag^+^ ion. [Ag^+^] = 5.0 × 10^–4^ M in tris buffer (pH = 8.0, final concentration = 10 mM). Due to the formation of the insoluble precipitate AgCl in tris-HCl buffer solution, the result of Ag^+^ was obtained after filtration of the turbid solution using a syringe filter (0.2 μm).


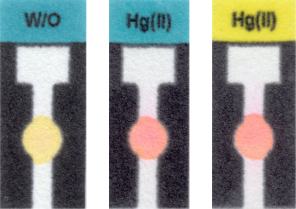


**Figure S14.** Effect of filtration on the Hg^2+^ signaling by the **RT**-equipped *μ*PAD kit. [Hg^2+^] = 5.0 × 10^–4^ M in tris-HCl buffer (pH = 8.0, final concentration = 10 mM). The result of Hg(II)-filtered was obtained after filtration of the solution using a syringe filter (0.2 μm).


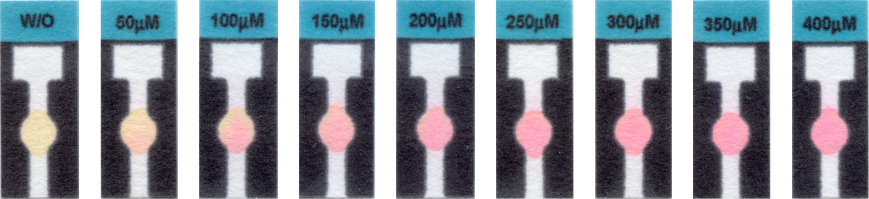


**Figure S15.** Changes in *Δ***L**_RG_ (**L**_red_ – **L**_green_) of **RT**-equipped *μ*PAD kit as a function of Hg^2+^ concentration ([Hg^2+^] = 0–4.0 × 10^–4^ M). Inset: Photograph of **RT**-equipped *μ*PAD kit in the presence of varying concentration of Hg^2+^ ion. [Hg^2+^] = 0–4.0 × 10^–4^ M in tris buffer solution (pH 8.0, final concentration = 10 mM).


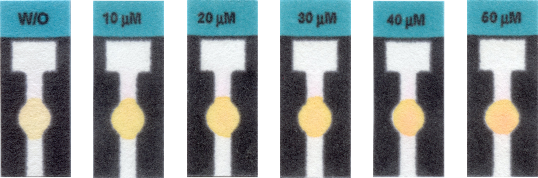


**Figure S16.** Changes in *Δ***L**_RG_ (**L**_red_ – **L**_green_) of the **RT**-equipped *μ*PAD kit as a function of Hg^2+^ concentration ([Hg^2+^] = 0–5.0 × 10^–5^ M). Inset: Photograph of the **RT**-equipped *μ*PAD kit in the presence of varying concentration of Hg^2+^ ion. [Hg^2+^] = 0–5.0 × 10^–5^ M in tris-HCl buffer solution (pH 8.0, final concentration = 10 mM).

**Figure S17.** Changes in *Δ***L**_RG_ (**L**_red_ – **L**_green_) of **RT**-equipped *μ*PAD kit as a function of Hg^2+^ concentration in simulated wastewater sample. Inset: Photograph of **RT**-equipped *μ*PAD kit in the presence of varying concentration of Hg^2+^ ion. [Hg^2+^] = 0–1.5 × 10^–4^ M in tris buffer solution (pH 8.0, final concentration = 10 mM).


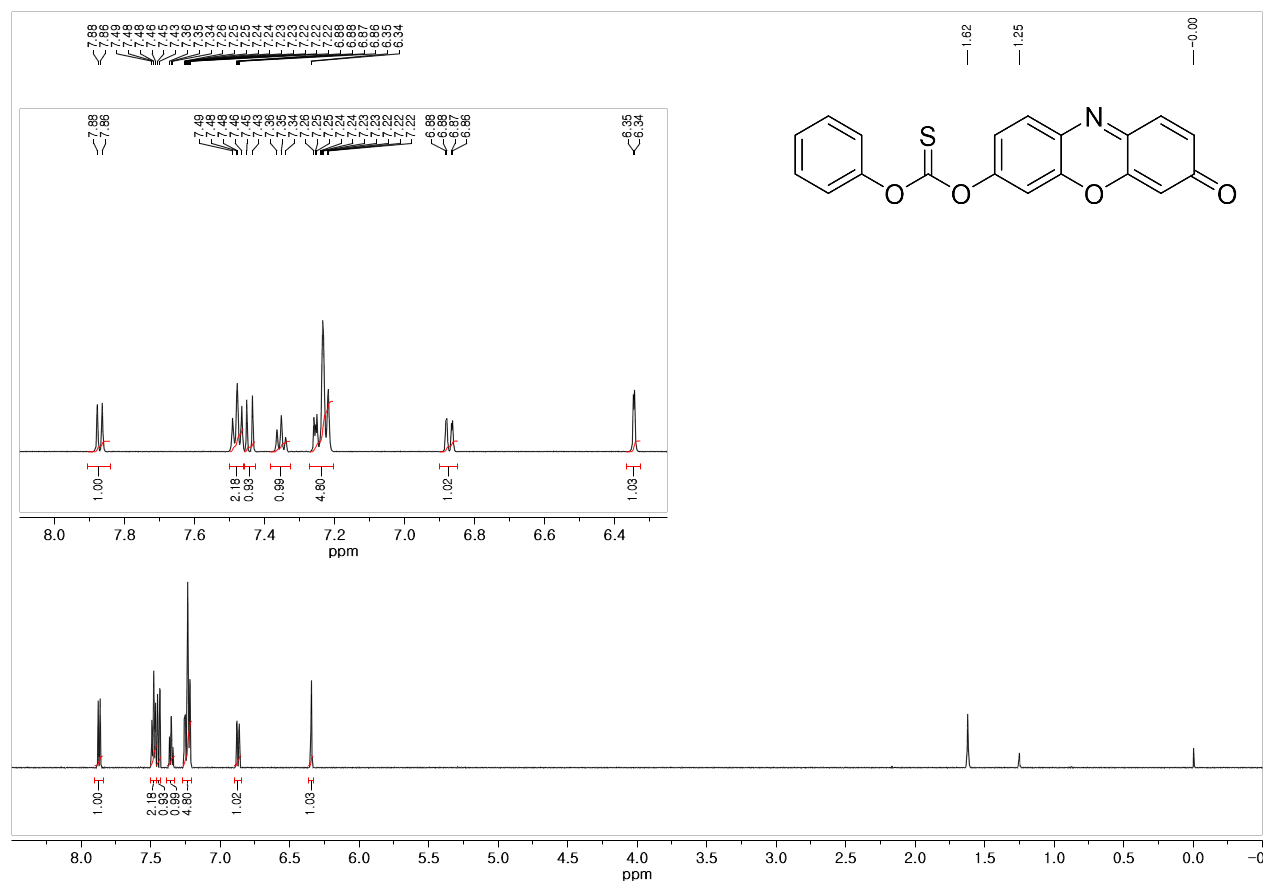


**RT**

**Figure S18.** ^1^H NMR spectrum of **RT** in CDCl_3_ (600 MHz).


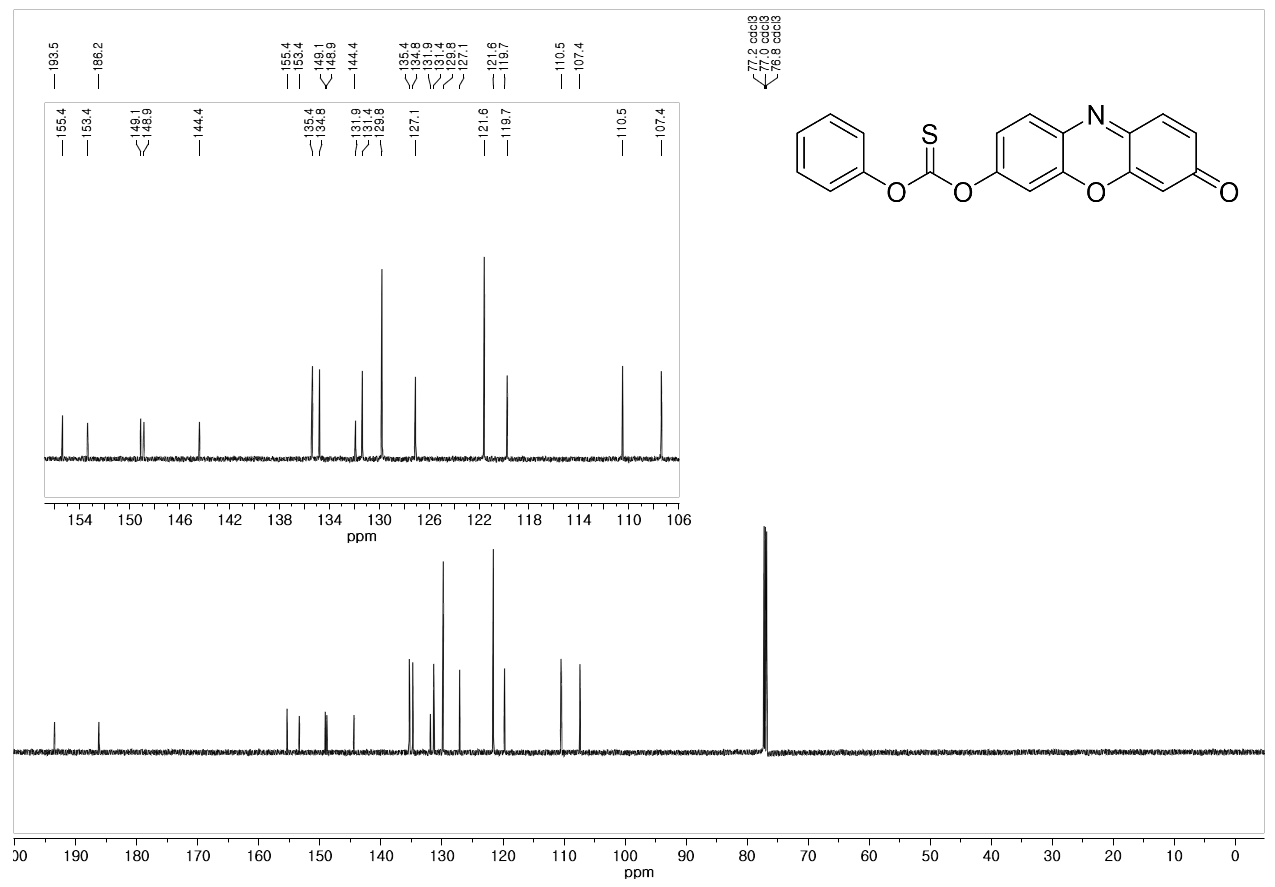


**RT**

**Figure S19.** ^13^C NMR spectrum of **RT** in CDCl_3_ (150 MHz).

**
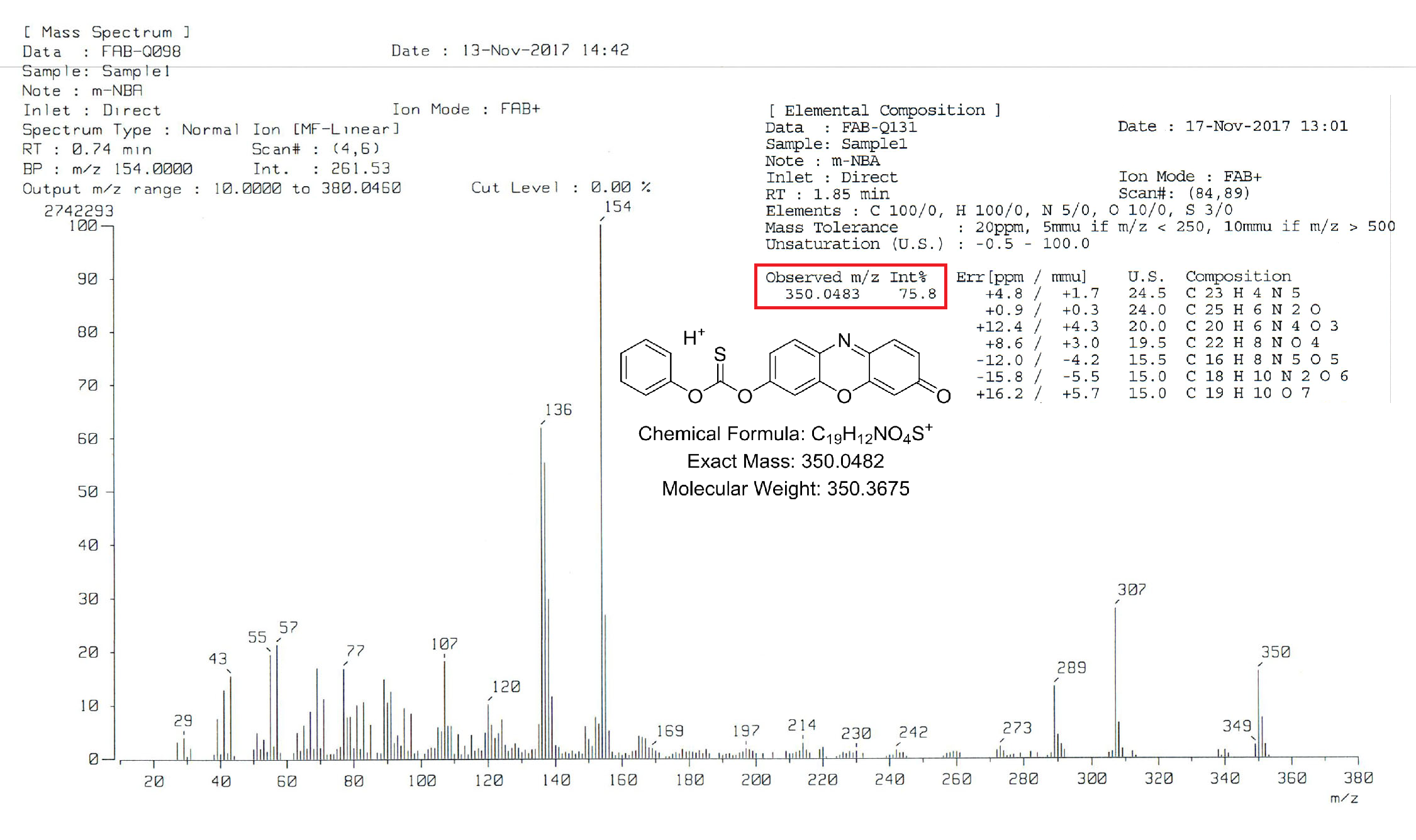
**

**Figure S20.** High-resolution FAB mass spectrum of **RT**.

References

[S1] Yuan, L., Lin, W. & Yang, Y. A ratiometric fluorescent probe for specific detection of cysteine over homocysteine and glutathione based on the drastic distinction in the kinetic profiles. *Chem. Commun.* **47**, 6275–6277 (2011).

[S2] Liu, C., Wu, H., Wang, Z., Shao, C., Zhu, B. & Zhang, X. A fast-response, highly sensitive and selective fluorescent probe for the ratiometric imaging of nitroxyl in living cells. *Chem. Commun.* **50**, 6013–6016 (2014).
